# Optimisation of a PC12 cell-based in vitro stroke model for screening neuroprotective agents

**DOI:** 10.1038/s41598-021-87431-4

**Published:** 2021-04-14

**Authors:** PinFen Chua, William K. Lim

**Affiliations:** Department of Paraclinical Sciences, Faculty of Medicine and Health Sciences, Universiti Malaysia Sarawak, 94300 Kota Samarahan, Sarawak Malaysia

**Keywords:** Drug discovery, Neuroscience

## Abstract

Stroke causes death and disability globally but no neuroprotectant is approved for post-stroke neuronal injury. Neuroprotective compounds can be identified using oxygen glucose deprivation (OGD) of neuronal cells as an in vitro stroke model. Nerve growth factor (NGF)-differentiated PC12 pheochromocytoma cells are frequently used. However, investigators often find their clonal variant undifferentiable and are uncertain of optimal culture conditions. Hence we studied 3 commonly used PC12 variants: PC12 Adh, PC12 from Riken Cell Bank (PC12 Riken) and Neuroscreen-1 (NS-1) cells. We found DMEM the optimal media for PC12 Riken and NS-1 cells. Using a novel serum-free media approach, we identified collagen IV as the preferred adhesive substrate for both cell lines. We found PC12 Adh cells cannot attach without serum and is unable to differentiate using NGF. NS-1 cells differentiated to a maximal 72.7 ± 5.2% %, with substantial basal differentiation. We optimised differentiated NS-1 cells for an in vitro stroke model using 3 h of OGD resulting in ~ 70% viable cells. We screened 5 reported neuroprotectants and provide the first report that serotonin is antiapoptotic in a stroke model and the 5-HT_1A_ agonist 8-hydroxy-2-(di-n-propylamino) tetralin (8-OH-DPAT) is neuroprotective in PC12 cells. Thus we demonstrate the optimisation and validation for a PC12 cell-based in vitro stroke model.

## Introduction

The PC12 pheochromocytoma cell line of neoplastic chromaffin cells was established from a rat adrenal tumour in 1976^1^. Treatment of these cells with nerve growth factor (NGF) stops its proliferation and promotes extension of fine, branching processes. Although not neurons, they were initially used for studying NGF signaling and neuronal differentiation^2^ and as an in vitro cytotoxicity model^3,4^. They are frequently used for in vitro identification of neuroprotectants against a variety of insults including serum deprivation^5^, toxic molecules^6,7^ and models of neurodegenerative diseases^8–10^.

Stroke or cerebrovascular ischaemia is a leading cause of death and disability worldwide, contributing significantly to long term health care costs^11^. Within minutes of cerebral vessel occlusion, neurons in the infarct core undergo necrotic cell death^12^. However, in the surrounding ischaemic penumbra the hypoperfused neurons, which may undergo apoptosis over hours to days, can be salvaged with appropriate intervention^13^. The current acute treatments for ischaemic stroke (intravenous thrombolysis and endovascular thrombectomy) only benefit less than 15% of stroke patients because they have to be administered within 4.5 and 6 h respectively from the onset of symptoms^14^. There is still no neuroprotective agent approved for inhibiting cell injury processes in the penumbra following acute cerebral ischaemic insult and/or reperfusion injury^15^.

Neuronal activity requires a continuous supply of oxygen and glucose^16^. Oxygen–glucose deprivation (OGD) mimics the pathophysiology of cerebral ischaemia and stroke^17^. PC12 cells are one of the most frequently employed neuronal cell lines for in vitro stroke models in the initial screening for neuroprotective compounds^18^. Clonal cell lines derived from neuronal tumours and immortalised in vitro allow an unlimited supply of homogenous cells which are transferable between laboratories^19^. Neuroprotectant drug properties demonstrated in such models can be validated using in vivo rat models^20,21^. Hence laboratories working with in vitro stroke models frequently use PC12 cells.

However, 30 years after the establishment of this cell line, variants were observed that lost NGF responsiveness and other desired traits^2^. A comparison of PC12 cells from 4 different laboratories revealed differences in morphology, adherence, enzyme expression, NGF responsiveness and requirements for media and substratum^22^. This extreme variability between PC12 cells of different laboratories is such that it no longer represents a single model and each investigator must screen the different derivations to select the most suitable one and its optimal growth conditions. This represents a major problem because many laboratories procure the cells from commercial suppliers.

We and others (K. Skieterska, personal communication) were unable to differentiate (using NGF) PC12 Adh (from ATCC), a well-known PC12 variant. It was evident from online fora^23^ that investigators often expended considerable time and resources for lack of guidance in the selection of commercial PC12 cell variants^24^. To address these uncertainties among PC12 cell users, we aim to examine 3 commonly used PC12 variants for their suitability as in vitro stroke models and then to optimise and validate one variant for identifying neuroprotective compounds. They are PC12 Adh, PC12 from Riken Cell Bank (PC12 Riken) and Neuroscreen-1 (NS-1) cells. These are commercially available, with the exception of NS-1 cells which was recently discontinued. We show the optimal media and substratum requirements for these PC12 variants. PC12 Adh produced negligible differentiation by NGF. In contrast, ~ 75% of PC12 NS-1 cells could be differentiated. Using NS-1 cells as an in vitro stroke model, we found 3 h of OGD optimal for screening neuroprotectants. We validated our PC12 cell-based model by showing the neuroprotective 5-HT_1A_ receptor agonist 8-hydroxy-2-(di-n-propylamino) tetralin (8-OH-DPAT) but not antagonist WAY100635 significantly reduced OGD-invoked apoptotic cell death and increased cell viability. Screening of 5 reported neuroprotective agents identified serotonin and fluoxetine as potential neuroprotective agents in ischaemic stroke.

## Results

### DMEM is the optimal media for PC12 Riken and NS-1 cells

**Figure 1:**
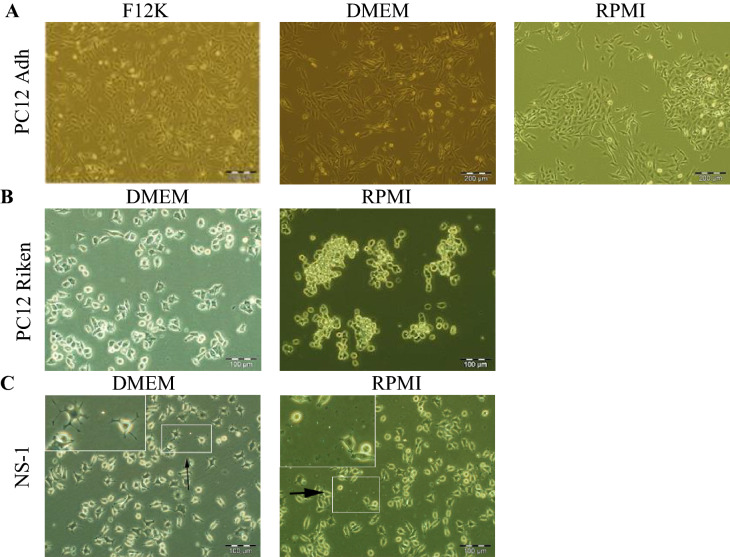
Optimisation of media for 3 PC12 cell variants. Cells were adapted from their original media as described in “Materials and methods”. Representative phase contrast images are shown after 3 passages in the new media and imaged 2–3 days after passaging. All cells were supplemented with 15% horse serum and 2.5% foetal bovine serum. (**A**) PC12 Adh cells were adapted from Ham’s F-12K to DMEM and RPMI, with no differences seen in cell morphology. (**B**) PC12 cells Riken were adapted from DMEM to RPMI resulting in rounded, phase-bright cells with loss of cell-substratum adhesion. (**C**) NS-1 cells adapted from RPMI to DMEM showed increased neurite outgrowth. Scale bar (**A**) 200 µm. (**B**,**C**) 100 µm.

Currently, the most commonly reported media for the culture of PC12 cells are DMEM and RPMI 1640. We aimed to adapt 3 PC12 variants into each of these 2 media to identify the optimal media for each variant. PC12 Adh is recommended by ATCC to be cultured in Ham’s F-12 K. We aimed to compare the morphology when the cells were adapted to DMEM and RPMI. PC12 Adh cells are a mix of polygonal and flattened, longish cells. As can be seen in Fig. 1A, virtually all the cells retained this morphology in all 3 media. Ham’s F-12 K medium was chosen for subsequent experiments as it was recommended by the supplier of the cells. Riken Cell Bank recommends DMEM for the culture of PC12 Riken cells. In DMEM, approximately three-fourths of the cells were polygonal shaped and formed clumps (Fig. 1B). We adapted it to RPMI and found nearly all the cells rounded and mostly unattached. Thus we cultured PC12 Riken in DMEM. The media recommended (by Cellomics) for NS-1 cells is RPMI. In our hands, the cells in RMPI showed minimal neurite extensions with about a third of them rounded, while debris and floaters were observed (Fig. 1C, inset panels). Hence we selected DMEM (only approximately a tenth rounded, no cell debris) as the media for NS-1 cells.

### Collagen type IV is the optimal substratum for PC12 Riken and NS-1 cells

**Figure 2:**
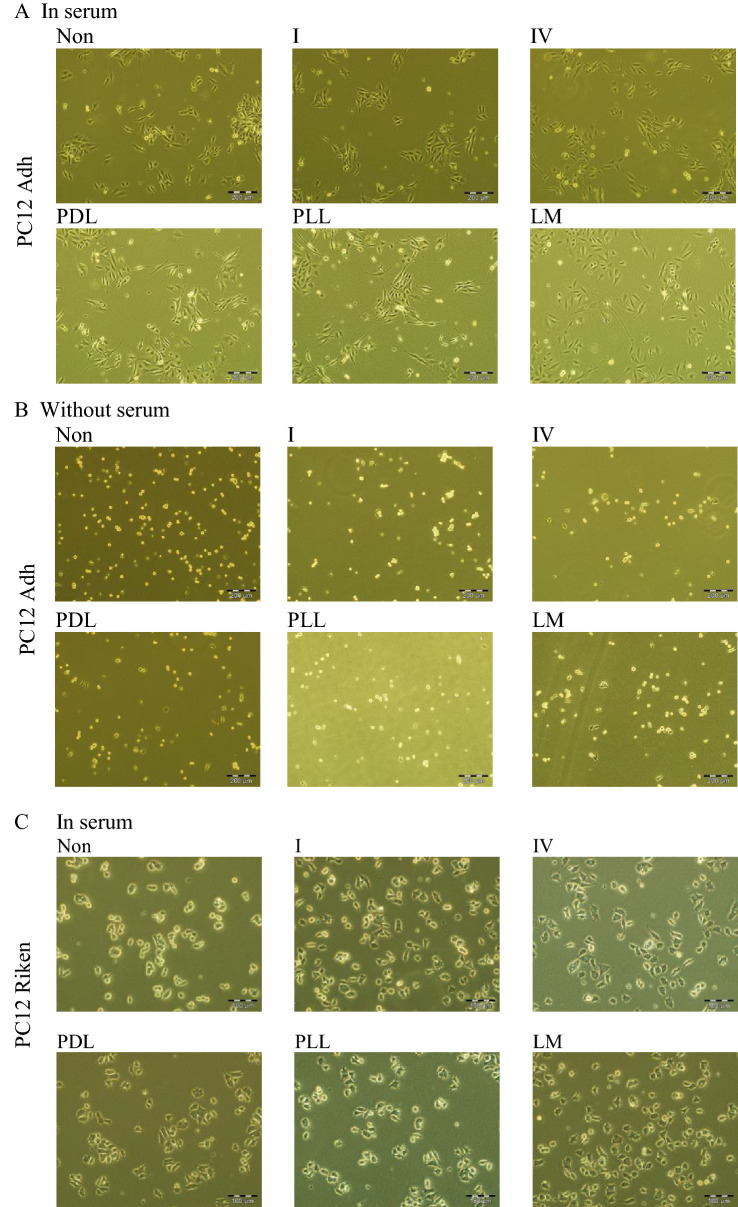

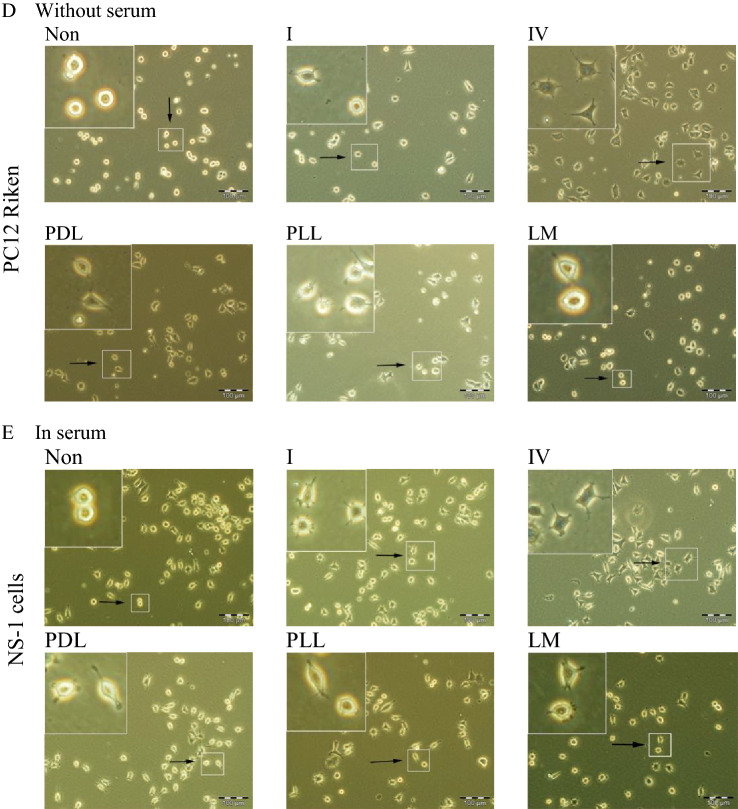
Optimisation of substratum coating for 3 PC12 variants. PC12 cells were cultured in media with 15% horse serum and 2.5% foetal bovine serum (**A**,**C**,**E**) or serum-free supplemented media (**B**,**D**) as described in “Materials and methods”. Cells were passaged into 6-well plates with the wells either uncoated (non) or coated with collagen I (I), collagen IV (IV), poly-d-lysine (PDL), poly-l-lysine (PLL) or laminin (LM). Representative phase contrast photomicrographs were imaged after 24 h. (**A**,**B**) PC12 Adh cells cultured in Ham’s F-12 K medium were passaged into a 6-well plate with the indicated substratum coating with serum (**A**) or without serum (**B**). (**C**,**D**) PC12 Riken cells cultured in DMEM medium were passaged into wells with the indicated substratum coatings with serum (**C**) or without serum (**D**). (**E**) NS-1 cells cultured in DMEM medium with serum were passaged into a 6-well plate having substrata with the indicated coatings. Scale bar (**A**,**B**) 200 µm. (**C**–**E**) 100 µm.

Anchorage-dependent cells require tissue culture surfaces to be appropriately treated or coated with an adhesive protein in order to be attached. Neurite outgrowth of PC12 cells is also highly dependent on the type of adhesive protein on the substrate^25^. Hence we wanted to determine the optimal substrata for culture of the 3 PC12 cell variants by comparing three extracellular matrix proteins (collagen types I and IV, and laminin) and two synthetic compounds that enhance cell adhesion (poly-d-Lysine and poly-l-Lysine). Figure 2A shows the morphology of PC12 Adh cells were similar across uncoated and all differently-coated wells alike: almost all cells were flat and spiky in uncoated and collagen-coated wells, while only about a tenth of cells were rounded in the other wells. Essentially the culture of PC12 Adh cells did not require precoating of tissue culture plasticware. Hence in the presence of serum, PC12 Adh cells did not distinguish between different substrata. One of the major functions of serum in culture media is to provide attachment and spreading factors to facilitate cell attachment to the substratum^26^. Hence cells cultured in serum-free media may require an adhesion substrate to remain attached. It follows that culture of PC12 cells in serum-free media may reveal its preferential adhesion substrate. Hence we tested the culture of PC12 Adh cells in serum-free supplemented media as described in “Materials and methods”. When cultured without serum (Fig. 2B), virtually all the cells were rounded, phase-bright and unattached. Therefore PC12 Adh cells in the absence of serum could not attach to the 5 substrata and hence we are not able to determine if Adh cells had a preferred substratum. For PC12 Riken cells, in uncoated wells only about a quarter of the cells were spread out, whereas in all the other coated substrata, the vast majority were well spread out (Fig. 2C). However, no one substratum gave a distinct advantage. Hence using this approach we were not able to identify an optimal substratum for PC12 Riken cells. Therefore we repeated this experiment in the absence of serum. (Fig. 2D). Figure 2D shows that PC12 Riken cells distinguished between different substrata when cultured without serum. In uncoated wells, all the cells were rounded, small and unattached. In wells coated with collagen type IV, 90.3 ± 2.0% cells were spread-out and phase-dark (see inset panel). In contrast, only 16.3–33.0% of cells in the other coated wells were spread out, with the majority being smaller, rounder and phase-bright. Hence collagen IV is optimal for PC12 Riken cells.

For NS-1 cells grown on collagen IV-coated wells, 67.7 ± 15.0% were flattened and spread-out (Fig. 2E, inset panel). In contrast, the next most effective substrata was PDL where only 31.5 ± 4.8% of the cells were spread-out. In the other substrata, the vast majority of cells were smaller, less spread and phase-bright. Hence collagen type IV was chosen for culture of NS-1 cells.

### NGF induces neurite outgrowth to the greatest extent in NS-1 cells

**Figure 3:**
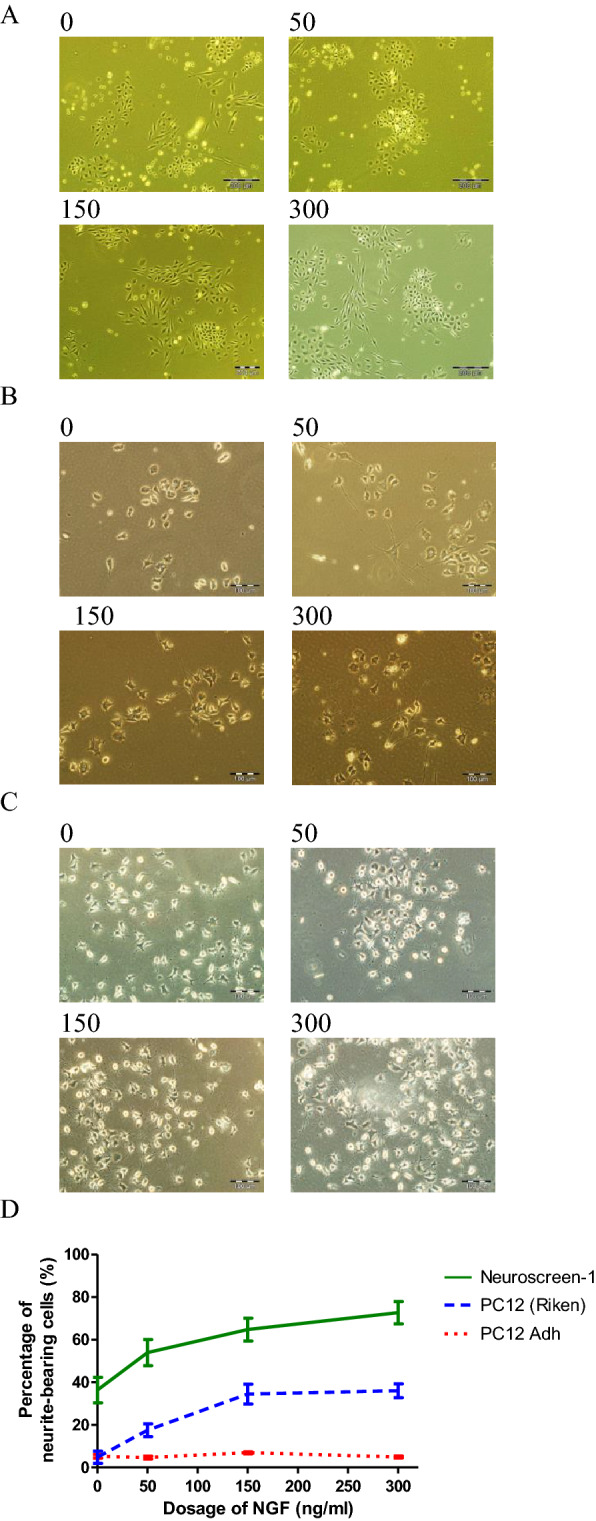
NGF differentiation of 3 PC12 cell variants. PC12 cells were cultured as described in “Materials and methods” and seeded into multiwell plates. After an overnight incubation, the media was changed to one with 2% horse serum (1% for PC12 Adh) and NGF added to the indicated concentration (ng/ml). The media and NGF was refreshed every 48 h and neurite scoring performed at 96 h. (**A**) PC12 Adh cells cultured in Ham’s F-12 K with serum were seeded into a poly-d-lysine-coated 6-well plate at 3 × 10^3^ cells/cm^2^. Representative phase contrast images at 100 × magnification were taken 96 h after addition of the indicated concentration of NGF (ng/ml). (**B**) PC12 Riken cells cultured in DMEM with serum were plated onto a 12-well plate coated with collagen IV at a density of 4 × 10^3^ cells/cm^2^. Representative phase contrast images at 200X magnification are shown 96 h after addition of NGF at the indicated concentrations (ng/ml). (**C**) NS-1 cells cultured in DMEM with serum were seeded into a collagen-IV coated 12-well plate at a density of 4 × 10^3^ cells/cm^2^. Representative phase contrast images (200X magnification) are shown 96 h after addition of NGF at the indicated concentration (ng/ml). (**D**) Three PC12 variants were treated with NGF at the indicated concentrations and the percentage of neurite-bearing cells obtained after 96 h. Data shown is the mean and ± SEM of 3 independent experiments. Scale bar (**A**) 200 µm, (**B**,**C**) 100 µm.

In the presence of NGF, PC12 cells stop dividing and extend long neuronal-like processes. As there are clonal variants with loss of responsiveness to NGF, it is important to know the prevalence of this phenomenon among common commercially available PC12 variants. Hence we studied the concentration responsiveness to NGF of 3 commonly-used, mostly commercially available clonal variants, with neuritic outgrowth as the index for neuronal differentiation. In Fig. 3A, PC12 Adh cells appear as a mixture of polygonal and flattened, spiky cells having short neurites. There is a low fraction of neurite-bearing cells even in 0 ng/ml NGF. Increase of NGF concentration up to 300 ng/ml does not significantly increase the prevalence of neurite-bearing cells. In contrast, PC12 Riken cells were mostly in an undifferentiated state in 0 ng/ml NGF (Fig. 3B). However, a noticeable increase in cells bearing long, thin neurites can be seen at 50 ng/ml NGF. This is further increased with higher concentrations of NGF as the neurites can be seen contacting other neurites or cell bodies. In contrast to PC12 Riken cells, NS-1 cells have a substantial proportion of neurite-bearing cells even in 0 ng/ml NGF (Fig. 3C). The percentage of cells with neuritic extensions is dependent on NGF concentration. Hence photomicrographs indicate major differences in the extent of neurite outgrowth among the 3 PC12 variants. To illustrate graphically the differences between the 3 PC12 variants, Fig. 3D shows the NGF concentration response curves for the proportion of cells expressing neurites when cultured in up to 2% serum. It can be seen that PC12 Adh cells differentiated to the least extent, with below 10% of the cells expressing neurites longer than one diameter of the cell soma. In contrast, PC12 Riken cells did not spontaneously express neurites. Induction of neurite outgrowth was a function of NGF concentration up to 150 ng/ml NGF, corresponding to ~ 30% of cells extending neurites after 96 h of treatment. Increase of NGF to 300 ng/ml gave no further cellular response. For NS-1 cells, almost 40% could extend neuritic processes without added NGF. This was statistically different *(p* < 0.01) from both PC12 Riken (36.4 ± 6.0% vs 4.8 ± 2.9%) and PC12 Adh cells (36.4 ± 11.9% vs 5.3 ± 0.9%). In the presence of NGF, the further increase of cells bearing neurites is concentration-dependent, reaching ~ 35% at 300 ng/ml NGF. Hence NS-1 cells could differentiate to the greatest extent, and we selected it for use as an in vitro stroke model. As there was a large overlap and no significant difference between cellular response to 150 and 300 ng/ml NGF, 150 ng/ml was selected as the optimal NGF concentration.

### Three hours of OGD in NS-1 cells is optimal for identifying neuroprotective compounds

**Figure 4:**
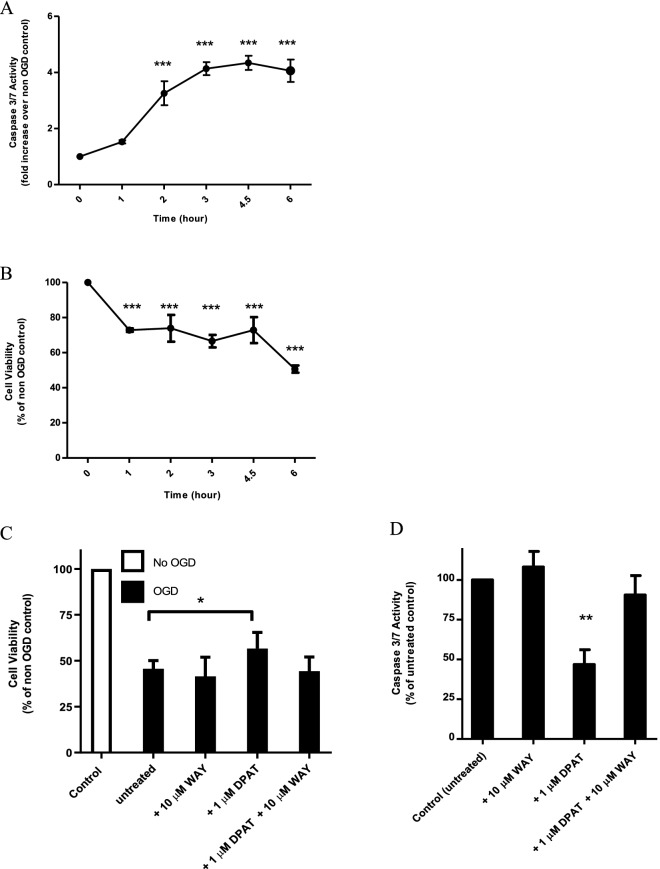
Optimisation and validation of PC12 NS-1 in vitro stroke model. (**A**) NGF-differentiated NS-1 cells were subjected to OGD as described under “Oxygen glucose deprivation”. Caspase 3/7 activity was measured at the indicated durations of OGD and data normalised as fold increase over cells without OGD. Data shown is mean ± SEM from 3 independent experiments of triplicate determination. Statistical testing was performed with one-way ANOVA with Dunnett’s post hoc test. ***p < 0.001 compared to non-OGD control (**B**) NS-1 cells differentiated with NGF were subjected to OGD as described under “Oxygen glucose deprivation” for the indicated durations. Cell viability was then assessed using the MTT assay and data normalised to control cells without OGD. Data are mean ± SEM of 3 independent experiments carried out in triplicate. ***p < 0.001 compared to non-OGD control. Differentiated NS-1 cells were subjected to 3 h OGD then 24 h of normal culture conditions added with WAY100635 or 8-OH-DPAT (or both) as described under “Drug treatment”. Subsequently, cell viability or apoptosis was measured. (**C**) Cell viability was determined with MTT assay. *p < 0.05 compared to non-OGD control. (**D**) Caspase 3/7 activity was measured. Non-OGD baseline activity was removed and data normalised to untreated cells. **p < 0.01 compared to untreated control. All data are expressed as mean ± SEM of 3 experiments determined in triplicate.

Oxygen glucose deprivation (OGD) mimics the cellular death observed in in vivo models of brain ischaemia. OGD-induced cell death in PC12 cells is mainly via apoptosis^27^. As caspase-3 is a key mediator of apoptosis in animal models of ischaemic stroke^13^, we aimed to optimise the duration of OGD for the activation of caspase-3 activity. Figure 4A shows the time course (hours) of OGD for activation of caspase 3/7 activity. Statistically significant difference (*p* < 0.001) was observed from 2 h onwards. As the extent of caspase 3/7 activation did not increase beyond 3 h of OGD, 3 h was selected as the optimal duration for OGD. For an OGD-based in vitro stroke model, cell death by OGD of 20–60% is ideal for assessing the neuroprotective effects of compounds^28^. Hence we utilised the MTT assay, a quantitative and reliable colorimetric assay for cell viability, to study the time course of OGD for reduction in cell viability (Fig. 4B). The percentage of viable cells was reduced to a statistically significant extent starting from the first hour. At 3 h, the proportion of dead cells was ~ 30% which is inside the range of cell death determined by Tabakman, Lazarovici and Kohen within which neuroprotective effects can be distinguished^28^.

### Optimised NS-1 cell-based in vitro stroke model is validated with 5-HT_1A_ receptor ligands

The treatment of stroke is carried out in a patient who already had the initial neurodegenerative insult. The OGD model is usually followed by a simulation of reperfusion in vivo when blood supply to the brain is re-established. Therefore, in the screening for potential neuroprotectants, the compound should be efficacious when given during the reoxygenation period after OGD. We intended to validate our optimised in vitro stroke model by treating with a known neuroprotectant during the reoxygenation period and investigate its effect on cell viability and apoptosis. The neuroprotectant 8-hydroxy-2-(di-n-propylamino) tetralin (8-OH-DPAT) is an agonist at 5-HT_1A_ receptors. To assess the ability of the model to distinguish between drugs of the same class, we tested this together with the antagonist N-[2-[4-(2-methoxyphenyl)-1-piperazinyl]ethyl]-N-2-pyridinyl-cyclohexanecarboxamide (WAY100635) which will bind the same receptors but produce no effect^29^. Both 1 μM 8-OH-DPAT and 10 μM WAY100635 were not toxic to non-OGD PC12 cells (data not shown). As can be seen in Fig. 4C, after 3 h of OGD and 24 h of reoxygenation, PC12 cell viability as measured by the MTT assay was 45.6% of non-OGD cells. However, the 5HT_1A_ receptor agonist 8-OH-DPAT (1 μM) significantly reduced cell death from 54.4 to 43.4% (p < 0.05). This increase in viability was abolished in the presence of 10 μM antagonist WAY10063, which had no effect on its own. To validate the ability of 8-OH-DPAT to attenuate OGD-induced apoptosis, caspase 3/7 activity was measured as the readout. Figure 4D showed 10 μM WAY100635 had no antiapoptotic effect but 1 μM 8-OH-DPAT reduced caspase 3/7 activity significantly (*p* < 0.01) by 53.2%. The action of 8-OH-DPAT was mediated by 5HT_1A_ receptors as it was abrogated by 10 μM of the antagonist WAY100635.

### Optimised in vitro stroke model is a useful screen for neuroprotective compounds in stroke

**Figure 5:**
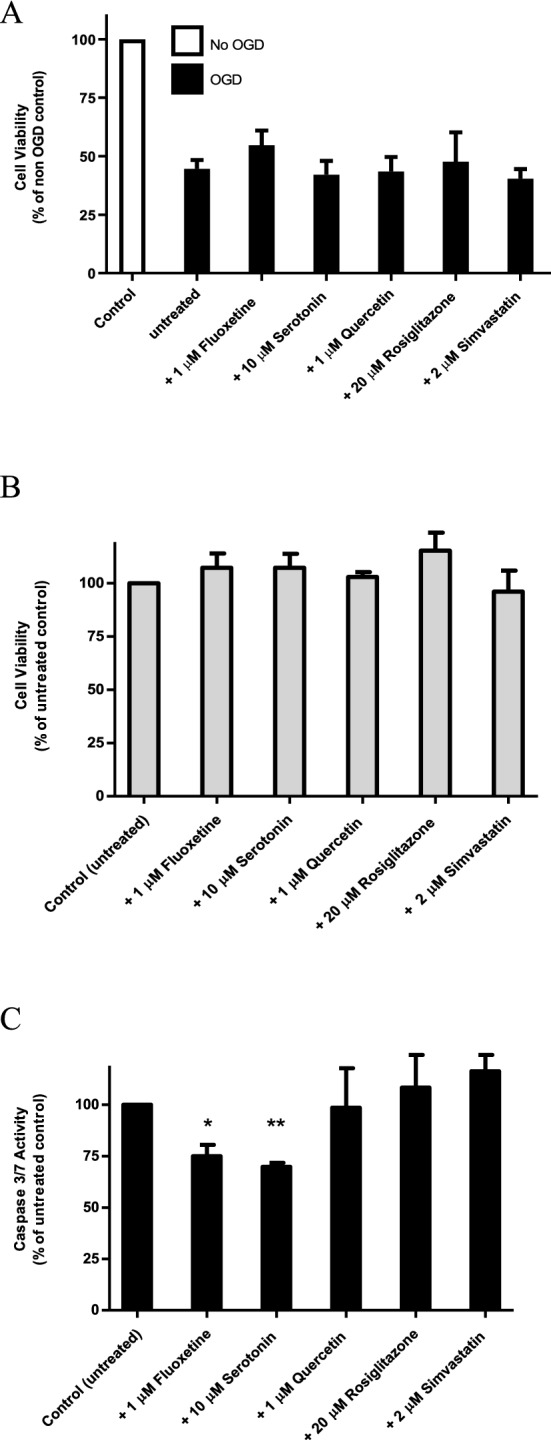
Application of PC12 NS-1 in vitro stroke model. (**A**) After 3-h OGD, NGF-differentiated NS-1 cells were incubated at normal conditions and treated with the indicated drugs as described under “Drug treatment”. Cell viability was determined by MTT assay. Data was normalised to untreated control cells. (**B**) Differentiated, non-OGD PC12 NS-1 cells were treated with the indicated drugs as described under “Drug treatment”. Cell viability was measured with MTT assay. Data was normalised to untreated control. (**C**) NGF-differentiated NS-1 cells were subjected to 3-h OGD followed by 24 h incubation at normal conditions with the indicated drugs as described under “Drug treatment”. Caspase 3/7 activity was measured. Non-OGD baseline activity was removed and data normalised to untreated cells. *p < 0.05 compared to control untreated cells; **p < 0.01 compared to control untreated cells. All data are expressed as mean ± SEM of 3 experiments determined in triplicate.

To evaluate the effectiveness of the NS-1 cell-based in vitro stroke model for identifying potential neuroprotective drugs, we used it to screen 5 compounds with reported neuroprotective properties: fluoxetine, serotonin, quercetin, rosiglitazone and simvastatin. At the concentration range used (1–20 μM), the compounds were not toxic to non-OGD cells (data not shown). Figure 5A shows that after 3 h OGD and 24 h reoxygenation, cell viability as measured by MTT assay was 44.6% of control non-OGD cells. When the 5 compounds were tested for an effect on overall cell death after OGD/reoxygenation, fluoxetine gave a measurable reduction in cell death from 55.5 to 45.3% (p > 0.05). As a control for a direct effect on PC12 cells, we treated non-OGD cells with the 5 compounds and measured cell viability by the MTT assay. From Fig. 5B, the compounds had no significant direct proliferative effect. To test the potential of the compounds to retard apoptosis in hypoperfused neurons, we treated OGD cells with the 5 compounds. As shown in Fig. 5C, caspase 3/7 activity was reduced significantly (p < 0.05 and p < 0.01) by 1 μM fluoxetine (25.0%) and 10 μM serotonin (30.2%) respectively. Hence in contrast to cell viability, serotonin and fluoxetine significantly attenuated OGD-induced apoptosis.

## Discussion

PC12 cells respond to the neurotrophin nerve growth factor (NGF) by differentiating into a phenotype with neuritic processes^1^. It is among the most common cell lines used as in vitro stroke models to identify potential neuroprotectants. Compounds identified can be validated in primary neurons and animal models. PC12 cells are used in neurobiology studies because of their ability to differentiate to relatively high levels^30^. However, online fora and discussions by investigators in this field often mention the problem of PC12 variants that cannot be differentiated^24^. Hence the PC12 cells available commercially or shared between laboratories now cannot be assumed to possess the characteristics of the original canonical PC12 cells. Despite awareness that clonal variants of PC12 cells differ in morphology, NGF-responsiveness and optimal culture conditions^31–34^, there is no systematic study on the phenotypic features of common commercially available PC12 cells. Hence we investigated 3 widely used commercially sourced PC12 cell variants: PC12 cells from Riken Cell Bank, Adh (CRL-1721.1 from ATCC) and Neuroscreen-1 (NS-1) cells formerly from Cellomics. Non-adherent PC12 cells (CRL-1721) from ATCC had been previously studied^22,25,35^.

When changes in the characteristics of PC12 cells were first reported, the remedy proposed was to culture with the original media, sera and substrate in order to avoid selecting for clonal variants, especially those non-responsive to NGF^30^. However, the high number of clonal variants now means there is no one standard culture condition. Instead, PC12 cells that are newly obtained or purchased need to be examined to ensure the phenotype is appropriate for the work it is intended for. It is important to know the maximal extent of differentiation possible for one’s PC12 variant, especially if it is for an in vitro stroke model where the ability to differentiate is a key requirement. Although commercially supplied cells come with recommended culture conditions, in this report we show the need to optimise media and substrate. We optimised the culture conditions of 3 frequently used commercially available PC12 variants and demonstrate their variation in capacity for NGF-induced differentiation. We report the lack of differentiation with Adh cells and selected NS-1 cells for optimisation and validation as an in vitro stroke model.

The original PC12 clonal cells were reported to extend processes in 80% of the cells after 2 weeks of NGF treatment^1^. We found up to 75% of NS-1 cells could be differentiated after 4 days of NGF treatment, comparable to a previous report^36^. This is consistent with the report that NS-1 cells require a shorter time to reach maximal differentiation relative to another PC12 variant^37,38^. However, it is important to distinguish between neurite outgrowth at basal condition versus after exposure to NGF. In the absence of NGF, only 10–20% of the canonical PC12 cells regenerated neurites^30^. We found comparable low levels of basal differentiation in PC12 Riken and PC12 Adh cells. In contrast, the basal differentiation of NS-1 cells was up to 40%. This could be the result of the interaction between cell adhesion molecules and the optimised coating substrate (collagen IV) which amplified signalling pathways for neuritic extension. Hence although NS-1 cells can reach a maximal level of differentiation (75%) comparable to the original PC12 cells, it is a combination of both basal (45%) and NGF-stimulated (35%) differentiation.

It was postulated that variant PC12 cell lines arose from spontaneous mutations^30^. The first reported NGF-insensitive PC12 variants were generated by mutagenesis^31^. PC12 Adh cells are often procured for its adherent phenotype, while ATCC provides no data on its differentiation. With the neurite scoring protocol we followed, PC12 Adh cells gave minimal differentiation of below 2% in response to NGF. In contrast, we showed PC12 Riken and NS-1 cells were able to differentiate up to ~ 30% and 35% respectively. This is consistent with personal communication and online fora discussion about the relative inability of PC12 Adh cells to be differentiated by NGF^24^. Differences observed in neurite outgrowth between PC12 (CRL-1721 from ATCC) and PC12 Adh cells^39^ have been attributed to differing signalling pathways in operation^40^. PC12 cell variants generated by mutagenesis that had altered response to NGF included those that had loss of NGF receptors^31^. Hence the low differentiation in Adh cells could be due to lowered expression of NGF receptors or altered signal transduction pathways.

PC12 was originally cultured in RPMI 1640 medium^1^. Tischler, Powers and Alroy suggested that culturing with DMEM instead of RPMI 1640 caused increased cell flattening and cell-substratum adhesion due to the higher Ca^2+^ concentration of DMEM^2^. Interestingly, PC12 cells mutagenised to select for clonal variants unresponsive to NGF also resulted in clones that exhibited a flattened epithelial morphology^31^. It has been reported that the signs of spontaneously arising PC12 variants include flat, spiky, rapidly dividing and non-NGF responsive cells^30^. This was postulated to be due to mutations in structural or regulatory proteins of the cytoskeleton which subsequently affected expression of NGF receptors and neurite extension. Mutagenised NGF-insensitive PC12 variants were reported to change to a flat morphology after 10–30 generations in culture^31^. A neurotoxicity study found PC12 cells carried beyond 16 passages lost the ability to differentiate and displayed alteration in morphology^34^. We found PC12 Adh cells differentiated only to a minimal extent. When cultured under conditions recommended by ATCC, the cells were initially an even mixture of polygonal shaped and flattened cells. Gradually the flat cells appeared longish and spiky until eventually almost all the cells were flat and spiky. Interestingly, it was reported that NGF-insensitive PC12 clones generated by mutagenesis were flatter than their spherical parental PC12 cells in approximately equal numbers, but the flatter cells proliferated more rapidly, eventually taking over the culture^31^. This was corroborated by another report that PC12 cells from ATCC (subtype unspecified) had more rounded somas and less fibroblastoid shape during earlier passage, but in later passage were flat, heterogeneous and fibroblast-like^34^. The original canonical PC12 cells were either round or polygonal in shape^1^. However, some published photomicrographs show PC12 cells as flat and spiky^41,42^. A recent report showing polygonal-shaped PC12 Adh cells that could differentiate to some extent^39^ could be early-passage cells. High-passage PC12 Adh cells are likely to be poorly-differentiating and appear flat, spiky and fibroblast-like.

The original canonical PC12 cells adhered poorly to tissue culture plastic and grew mostly as floating cell aggregates. Attachment to plastic dishes was achieved by precoating the dishes with rat tail collagen^1^. The phenotype of a cell is influenced by both media and substratum^22^. The strength of its attachment to the substratum influences neurite initiation and outgrowth^32^ while the substratum also influences adhesion, growth and differentiation of cells^35^. But since the PC12 cell line is no longer a single uniform model, there is a need to determine the optimal substratum coating for each variant. Hence we investigated the optimal substratum for 3 commonly used, commercially available variants. We found Adh cells had no need for a substratum coating, as stated by ATCC, showing there are clonal variants that can adhere directly to tissue culture plastic. The supplier of NS-1 cells (Cellomics) recommended collagen I as the preferred substrate but we showed collagen IV (in DMEM media) to support better cell attachment for both NS-1 and PC12 Riken cells, the latter of which corroborated a previous report^43^. A study using the original canonical PC12 cells found collagen IV, poly-d-lysine and laminin to be equal for supporting attachment^44^ while a study in PC12 cells (ATCC) did not include collagen^35^.

Cell culture without serum is sometimes preferred to avoid differing results from different batches of sera. In this report, we show 2 additional reasons to culture without serum. First, we used serum-free media to determine the optimal adhesive substrate of PC12 Riken cells. PC12 Riken cell morphology was similar when cultured with different substrata in media containing serum. However, it is necessary to elucidate the optimal substrate as it can have a bearing on cell differentiation^25^. Our serum-free, supplemented media contains six supplements as previously reported^43^. As serum contains conditioning factors that aid in cell-substratum attachment^26^, the removal of serum causes cell attachment to be solely dependent on the substratum. Using this novel approach, we confirm collagen IV as the preferred substratum for PC12 Riken cells. We also provide the first report that in the absence of serum, PC12 Adh cells are rounded-up and detached, whereas NS-1 cells remain attached and viable. Hence PC12 Adh cells may be absolutely dependent on a factor in serum for their attachment. Second, serum-free media may be essential to isolate neuroprotective compounds where the same compounds or its analogues may be present in serum. Hence we tested potential neuroprotective compounds in serum-free, supplemented media, to enable their neuroprotective effect to be detected without interference from molecules in serum that either add to or subtract from that effect. This may be important if the compound of interest is of low concentration in a mixture with other compounds.

In vitro models of stroke such as OGD has enabled the study of cellular pathways associated with brain ischaemia and enabled identification of potential therapeutic agents. Insults to the brain that interrupt its blood or oxygen supply combined with a massive reduction in glucose lead to rapid neuronal death. We assayed OGD-invoked activity of caspase 3/7, being major effector enzymes in the execution phase of apoptosis^45^ that play a major role in hypoxia/ischaemia-mediated injury^46^. Without treatment, the penumbra can progress to infarction through excitotoxicity, spreading depolarisations, inflammation and apoptosis^47^. In contrast to cerebral occlusion, in vitro OGD causes cellular injury to occur over 1–4 hours^18^. Our stroke model is based on 3 h of hypoxia and hypoglycaemia followed by 24 h of reoxygenation. OGD is carried out without glucose, serum or serum supplements to reflect underperfusion of the penumbra during stroke. Media was not replaced after OGD in order to retain any factors released during OGD, as would occur in an in vivo setting^48^. To simulate the return of blood supply during reperfusion, we added serum supplements and glucose at the start of reoxygenation. Thus our model is useful for identifying neuroprotective agents to be administered in the hours to days following an ischaemic stroke. Compounds which can lower apoptotic cell death in the penumbra can potentially reduce secondary cerebral injury and disability.

We initiated drug treatment during reoxygenation to simulate the administration of neuroprotective drug after surgical, pharmacological or spontaneous recanalisation. Post-injury neuroprotective activity is necessary for validation of our in vitro stroke model^49^ as stroke patients present for treatment after the initial ischaemic insult^50^. 8-OH-DPAT has been shown to be neuroprotective in cultured cells^51^, primary neurons^52^ and in vivo settings^53,54^. Its EC_50_ for activation of 5-HT_1A_ receptors in the brain is below 100nM^55^. We found treatment with 1 μM 8-OH-DPAT significantly increased cell viability and decreased OGD-induced caspase 3/7 activity compared to no treatment. In stressed neuronal cells, 5-HT_1A_ receptors were found to mediate survival^56^. In contrast, WAY100635 binds selectively to 5-HT_1A_ receptors but does not activate the receptors^29^. It was found to block 5-HT-stimulated response with a IC_50_ of under 7nM^57^. We treated OGD cells with 10 μM WAY100635 and saw no increase in cell viability or anti-apoptotic effect. Furthermore, a tenfold higher concentration of WAY100635 abolished both the increase in cell viability and anti-apoptotic effect of 8-OH-DPAT, showing that the neuroprotective effect of 8-OH-DPAT is most likely mediated by activation of 5-HT_1A_ receptors. This is the first report that 8-OH-DPAT is neuroprotective in PC12 cells. It validates our NS-1 cell-based in vitro stroke model for the identification of neuroprotective compounds.

We evaluated our optimised NS-1 cell-based stroke model using 5 compounds reported to provide neuroprotection in different models. The effect of the 5 neuroprotective compounds on cell viability did not reach statistical significance. In our model, after 3-h OGD and 24-h reoxygenation, about 54% of the cells were no longer viable. This parallels the neuronal death caused by mechanisms such as necrosis (in the infarct core) and apoptosis (in the penumbra) after stroke. With such a high percentage of dead cells as baseline, any reduction in apoptotic cell death by the compounds is less likely to produce a significant increase in overall cell viability. In contrast to measuring total cell viability, apoptosis is but one of many mechanisms causing neuronal death, the blocking of which is known to be neuroprotective^58^. When apoptosis was directly measured, both serotonin (5-HT) and fluoxetine produced a significant reduction.

Serotonin is a neurotransmitter involved in most biological functions^59^. It has been found to be neuroprotective in models of Alzheimer’s^60^ and Parkinson’s disease^61^. This is the first report of serotonin having neuroprotective potential in a stroke model. It is likely to work through distinct serotonin receptors, just as serotonin enhances neuritogenesis in PC12 cells through 5-HT_3_ receptors^62^ and regulates mitochondrial biogenesis in cortical neurons via 5-HT_2A_ receptors^63^. Fluoxetine, a selective serotonin reuptake inhibitor that had entered clinical trial for stroke treatment^64^ is neuroprotective in neuronal and animal models. Its neuroprotective mechanism has been postulated to be independent of serotonin signalling^65,66^. The other 3 compounds did not display neuroprotective activity in our screen. Interestingly, 2 large screens of known neuroprotectants found only about half exhibited neuroprotective effect^49,67^. This was attributed to the use of different animal/cellular model, dose of drug and treatment protocol. We initiate drug treatment during re-oxygenation to better reflect clinical practice whereas other laboratories may treat before OGD, which leads to a higher rate of positive results. Quercetin has been found to be neuroprotective in various models of neuronal injury and neurodegenerative diseases^68^. However, it has also been reported to provoke apoptotic cell death in PC12 cells^69^. PPARγ agonists such as rosiglitazone have been shown to be neuroprotective in neuronal and animal models^70^. Nonetheless, it was also reported to stimulate apoptosis in PC12 cells^71^. Statins are in clinical trials for the prevention and management of stroke^72^ but there are also in vitro studies reporting its neurotoxicity^73^. Hence although drug screening systems have limitations, our in vitro stroke model is able to identify potentially useful neuroprotectants.

In conclusion, we have addressed the significant problem among neurobiologists regarding phenotypic differences among PC12 cell variants, which is of particular concern among laboratories using in vitro stroke models where the ability to differentiate is usually a key requirement. It is incumbent upon each laboratory who receive PC12 cells to ascertain its optimal media, adhesive substrate and maximal level of differentiation. Here we show the variation in differentiation capacity among commonly used PC12 cell lines, and report the lack of differentiation with PC12 Adh cells (ATCC). Using NS-1 cells as an in vitro stroke model, we found 3 h of OGD optimal for screening neuroprotectants. We validated our model by showing that the 5-HT_1A_ receptor agonist 8-hydroxy-2-(di-n-propylamino) tetralin (8-OH-DPAT) but not antagonist WAY100635 significantly reduced OGD-invoked apoptotic cell death. We screened 5 neuroprotective compounds with our in vitro stroke model and provide the first report that serotonin is a potential neuroprotectant in ischaemic stroke.

## Materials and methods

### Reagents and chemicals

Culture medium (RPMI 1640, DMEM, Ham’s F-12 K), horse serum, GlutaMax and bovine serum albumin were from Gibco (Grand Island, NY). Foetal bovine serum was from Hyclone (Logan, UT). Collagen I and collagen IV were from Advanced Biomatrix (Carlsbad, CA).

Poly-d-Lysine and poly-l-Lysine were from Merck (Burlington, MA). Poly-d-Lysine-coated 6-well plates were from Thermoscientific (Rochester, NY). 8-OH-DPAT, WAY100635 maleate, fluoxetine hydrochloride, rosiglitazone and simvastatin were from Tocris (Bristol, UK). Serotonin creatinine sulfate monohydrate, quercetin dihydrate and all other biochemicals were from Sigma Aldrich (St. Louis, MO).

### Cell culture

PC12 Adh (CRL-1721.1) cells were purchased from ATCC (Manassas, VA) and cultured in Ham’s F-12 K medium supplemented with 2.5% foetal bovine serum and 15% horse serum. PC12 (originator: L. Greene and A. Tischler) RCB0009 cells (Riken) were obtained from Riken Cell Bank (Tsukuba, Ibaraki, Japan). Neuroscreen-1 (NS-1) cells (formerly from Cellomics) were a generous gift from Dr Yves Le Dréan, University of Rennes 1, France. Both PC12 Riken and NS-1 cells were cultured in DMEM supplemented with 2.5% foetal bovine serum and 15% horse serum. Cell cultures were incubated in a chamber at 37 °C under 5% CO_2_. Cells used for experiments were those that had been passaged from between 3 and 13 times.

### Adaptation to new medium

Cells were adapted to new media essentially following the protocol of the ATCC Animal Cell Culture Guide (https://www.atcc.org/~/media/PDFs/Culture%20Guides/AnimCellCulture_Guide.ashx). Briefly, one culture was maintained in the original culture medium as a control while another is progressively diluted into the new media (from 50 to 75%, 87.5% and 100%) with each passage. The confluence rate and morphology of both cultures were monitored for similarity until the adapting cells were totally in the new media. Each experiment was repeated three times. Cell morphology was visualised using brightfield phase contrast microscopy with an Olympus IX71 inverted microscope. In each experiment one field was randomly chosen and images captured with an Olympus DP72 camera then examined using Cell^F imaging software. All the cells (at least 50) in the image were counted.

### Adaptation to serum-free supplemented media

PC12 variants were cultured in media as described under “Cell culture”. Cells were adapted to new serum-free media essentially following the ATCC Animal Cell Culture Guide as described under “Adaptation to new medium” and adaptation was repeated three times. The serum-free media was modified from a published serum-free media for PC12 cells^43^ and consisted of the original media without serum but supplemented with 30 µg/mL insulin, 10 µg/ml apo-transferrin, 500 µg/ml BSA, 10 µM ethanolamine, 10 µM β-mercaptoethanol and 10 nM sodium selenite.

### Optimisation of substratum coating

The 3 PC12 variants were cultured in media as described under “Cell culture” and plated on 6-well plates uncoated, or coated with 90 µg/ml collagen Type I, 10 µg/ml collagen Type IV, 50 µg/ml poly-d-lysine, 50 µg/ml poly-l-lysine or 10 µg/ml laminin. After 24 h, the morphology of the cells were observed. In cell variants showing no observable differences, the procedure was repeated under serum-free conditions, where the cells were initially cultured in 75% serum-free supplemented media. The cells were then plated on 6-well plates with the different coatings as described above, in 100% serum-free supplemented media. Cellular morphology was observed 24 h later. All experiments were repeated three times.

### NGF differentiation

PC12 variants were cultured in media as described under “Cell culture”. PC12 Adh cells were seeded into multiwell plates coated with poly-d-lysine whereas PC12 Riken and NS-1 cells were seeded into collagen IV-coated multiwell plates. After an overnight incubation, the media was changed to one containing 1% horse serum (for PC12 Adh cells) or 2% horse serum (for PC12 Riken and NS-1 cells) and NGF added to the indicated concentration. The media and NGF was renewed every 48 h. After 96 h (or otherwise indicated), cells were scored for the percentage of neurite-bearing cells.

### Neurite scoring

Neurite scoring was performed on inverted phase contrast microscopic images. A neurite-bearing cell was defined as one having a neurite at least equal to its cell body diameter^74^. Neurite measurements were made using ImageJ 1.51 k software (NIH). The proportion of neurite-bearing cells was the number of neurite-bearing cells divided by the total number of cells counted. At least 150 cells were counted from at least 4 randomly chosen fields per well and the experiment repeated three times.

### Oxygen glucose deprivation

The medium of NGF-differentiated cells at ~ 60–70% confluence growing in 96-well plates was replaced with glucose- and serum-free medium. The plates were placed in a Becton Dickinson Bio-Bag (Cat nos 261216, Cockeysville, MD) anaerobic environmental chamber at 37 °C following manufacturer’s instructions^56^. The level of O_2_ inside the chamber is below 0.1% and CO_2_ at 8–12% for the indicated times.

### MTT assay

Cell viability was assessed using the MTT (3-(4,5-dimethylthiazol-2-yl)-2,5-diphenyltetrazolium bromide) tetrazolium assay. NS-1 cells grown in collagen-IV coated plates were differentiated with NGF and subjected to oxygen–glucose deprivation as described under “Oxygen glucose deprivation”. Subsequently the cells were incubated at 37 °C in a 5% CO_2_ incubator in medium containing glucose and MTT (0.5 mg/mL) for 3 h. The medium was then removed and 100 µL of DMSO added to each well. After mixing on an orbital shaker for 10–15 min, the absorbance was read at 570 nm using a Infinite 200 Pro microplate reader (Tecan, Switzerland).

### Caspase 3/7 activity assay

Caspase 3/7 activity was determined using the Caspase-Glo 3/7 Assay Kit (Promega, Madison, WI) according to the manufacturer’s instructions. Briefly, NS-1 cells grown in collagen-IV coated plates were differentiated with NGF and subjected to oxygen–glucose deprivation (OGD) at the indicated duration as previously described. Caspase-Glo 3/7 reagent was added to each well and the plate gently mixed at 400 rpm for 30 s followed by incubation at 25 °C for 30 min. Subsequently luminescence was measured using a Microbeta Lumijet microplate counter (PerkinElmer, Waltham, MA).

### Drug treatment

After 3 h of OGD, the plate was removed from the anaerobic chamber and the indicated drugs (8-OH-DPAT, WAY100635 maleate, fluoxetine hydrochloride, serotonin creatinine sulfate monohydrate, quercetin dihydrate, rosiglitazone and simvastatin) were added to each well. Glucose was added to each well to reach the level before OGD. The serum-free supplements listed under “Adaptation to serum-free supplemented media” were also added to each well. The plate was then incubated at 37 °C in a 5% CO_2_ incubator with normal oxygenation. After 24 h, cell viability was measured as described under “MTT assay” and caspase 3/7 activity was measured as described under “Caspase 3/7 activity assay”.

### Statistical analysis

All data was analysed using Prism 5 software (GraphPad, La Jolla, CA). Data sets were tested for statistical significance using Student’s t-test for comparing 2 groups of data or one-way ANOVA followed by post hoc Dunnett's comparison test for comparing 3 or more groups of data. Differences were considered statistically significant when p < 0.05. Data is reported as mean ± SEM of at least three independent repeats, each performed in triplicate.
